# The Potential Application of Human Gingival Fibroblast-Conditioned Media in Pulp Regeneration: An In Vitro Study

**DOI:** 10.3390/cells11213398

**Published:** 2022-10-27

**Authors:** Huong Thu Vu, Ji-Young Yoon, Jae-Hee Park, Hae-Hyoung Lee, Khandmaa Dashnyam, Hae-Won Kim, Jung-Hwan Lee, Ji-Sun Shin, Jong-Bin Kim

**Affiliations:** 1 Department of Pediatric Dentistry, College of Dentistry, Dankook University, 119 Dandae-ro, Cheonan 31116, Chungcheongnam-do, Korea; 2 Institute of Tissue Regeneration Engineering (ITREN), Dankook University, 119 Dandae-ro, Cheonan 31116, Chungcheongnam-do, Korea; 3 Cell & Matter Institute, Dankook University, 119 Dandae-ro, Cheonan 31116, Chungcheongnam-do, Korea; 4 Department of Nanobiomedical Science and BK21 PLUS NBM Global Research Center for Regenerative Medicine, Dankook University, 119 Dandae-ro, Cheonan 31116, Chungcheongnam-do, Korea; 5 Department of Biomaterials science, College of Dentistry, Dankook University, 119 Dandae-ro, Cheonan 31116, Chungcheongnam-do, Korea; 6 UCL Eastman-Korea Dental Medicine Innovation Centre, Dankook University, 119 Dandae-ro, Cheonan 31116, Chungcheongnam-do, Korea; 7 Drug Research Institute, Mongolian University of Pharmaceutical Science, Ulaanbaatar 976, Mongolia; 8 Mechanobiology Dental Medicine Research Centre, Dankook University, 119 Dandae-ro, Cheonan 31116, Chungcheongnam-do, Korea

**Keywords:** conditioned medium, gingival fibroblast, human exfoliated deciduous teeth, odontoblast differentiation, pulp regeneration

## Abstract

Regenerative endodontic treatment based on tissue engineering has recently gained interest in contemporary restorative dentistry. However, low survival rates and poor potential differentiation of stem cells could undermine the success rate of pulp regenerative therapy. Human gingival fibroblast-conditioned medium (hGF-CM) has been considered a potential therapy for tissue regeneration due to its stability in maintaining multiple factors essential for tissue regeneration compared to live cell transplantation. This study aimed to investigate the potency of hGF-CM on stem cells from human dental pulp (DPSC) in pulp regeneration. A series of experiments confirmed that hGF-CM contributes to a significant increase in proliferation, migration capability, and cell viability of DPSC after H_2_O_2_ exposure. Moreover, it has been proved to facilitate the odontogenic differentiation of DPSC via qRT-PCR, ALP (alkaline phosphatase), and ARS (Alizarin Red S) staining. It has been discovered that such highly upregulated odontogenesis is related to certain types of ECM proteins (collagen and laminin) from hGF-CM via proteomics. In addition, it is found that the ERK pathway is a key mechanism via inhibition assay based on RNA-seq result. These findings demonstrate that hGF-CM could be beneficial biomolecules for pulp regeneration.

## 1. Introduction

Pulpal disease is one of the most prevalent oral diseases in humans, and it is the main cause of tooth loss in young people [1,2]. For the treatment of this disease, root canal treatment is routinely performed, which involves the removal of contaminated dental pulp tissue and the replacement of the tissue with inert, biocompatible materials [3]. Although this approach has been confirmed to be extremely effective, with a decent prognosis and high success rates [4,5], it often restricts regeneration in immature teeth with incomplete root development [6]. Apexification is a conventional method to induce apical closure in immature permanent teeth in which root formation is halted due to pulp necrosis [6,7,8,9]. However, the technique does have limitations: treated teeth may develop complications such as thick dentinal walls, tooth discoloration, or biointeraction of the obturation material when it is maintained for a long time [10,11,12]. To solve this problem, an alternative treatment to apexification has been developed in pulp regenerative therapy. Since the early 2000s, pulp revascularization has been attempted as a novel therapeutic treatment and has brought success in the induction of angiogenesis and apexification [7,13]. Nevertheless, pulp regeneration in its truest definition requires the involvement of the odontoblastic layer lining beneath the dentin surface, a phenomenon that is still often absent in current practices. Moreover, histological staining revealed that newly formed tissue in the canals was nonpulp-like tissues consisting of cement, bone-like mineral, and periodontal-like connective tissue in most revascularization cases [13,14,15]. Therefore, pulp regeneration studies have shifted toward stem cell-based regenerative therapy, the main goal being the regeneration of vital pulp-like tissue.

Cell-based pulp-like tissue regeneration is related to the different and multiple interactions between stem/progenitor cells with specific signal transduction to be seeded on biocompatible scaffolds placed into root canals [16]. For the main role, stem cells (MSCs) are favored due to their regeneration potentials and multiple lineage differentiation. Among well-known MSCs, dental stem cells have been considered a potentially fertile source of MSCs for the study of cell-based pulp regeneration after Gronthos and Muira reported success in the culture of dental pulp stem cells from permanent teeth (DPSCs) and exfoliated deciduous teeth (SHEDs) [17,18].

One of the key issues in pulp regeneration is that transplanted cells in the root canal should survive in a hypoxic/inflammatory environment before neovascularization. Many studies aim to create a favorable environment that can promote cell recruitment, cell proliferation, and differentiation [16]. Several studies have also found epigenetic modifications influence several critical signal pathways, controlling DPSC’s characterization, such as multipotency and self-renewal capacity. However, these epigenetic therapeutics remain more investigated to overcome unwanted effects, the possibility of neoplastic transformation, etc., for clinical application [19]. Another line of research to successfully achieve this goal is the usage of conditioned media. In particular, conditioned media derived from cultured mesenchymal stem cells contain various secreted cytokines, growth factors, extracellular matrix proteins, and miscellaneous factors involved in cellular proliferation and tissue regeneration [20,21,22,23,24,25,26]. In addition, these media can be used in cell-free therapy as an alternative to cell-based therapy, as the utilization can lower risks of immune rejection and reduce the possibility of allogenic administration and are relatively more feasible for commercial mass production. Thus, stem cell-derived conditioned media are promising prospects to be produced as pharmaceutical agents for regenerative medicine. Various dental stem cell-derived medium has been reported the possibility as a candidate for tissue regeneration in other literature [27]. In particular, Cara et al. reported that SHED-CM has angiogenic properties and induced tissue formation inside the root canal, and Xian et al. reported the possibility DPSC-Exo by proving the promotion of HUVEC proliferation, pro-angiogenic factor expression, and tube formation, and Hong et al. showed the rat DFSC (Dental follicle stem cells)-derived CM has great effects on repair of the inflamed dental pulp trough in vitro and in vivo tests [28,29,30]. However, those are less clinical because the tooth extraction should be accompanied to get the stem cells and are limited tissue volume. Among the various types of dental stem cells that could be selected for conditioned media manufacture, human gingival-derived mesenchymal stem cells or gingival fibroblasts (hGFs) originate from gingiva connective tissue have been considered an endless resource for mesenchymal stem cells due to the availability and accessibility of gingival tissues via a minimally invasive route treatment. Gingival fibroblasts show similar properties to mesenchymal stem cells-like cells (MSC), such as immunomodulatory properties and multipotency for differentiation into various mesenchymal lineage, and have been considered a useful candidate for application in anticancer therapy, skin repair, periodontal tendon regeneration, etc. [31,32,33,34]. Not surprisingly, extracellular vesicles (EVs) containing conditioned media collected from human gingival fibroblast (hGF) cultures have been reported to be effective in osteoblast differentiation, neuronal development, and angiogenesis [20,35]. In particular, members of the Wnt family, which are involved in the processes of cellular proliferation and tissue regeneration, are known to be highly expressed in vesicles. Moreover, the transcriptome profile of EVs of hGF-CM confirmed the presence of several interleukins, some of which can drive anti-inflammatory action [35]. Therefore, a number of studies have been conducted to investigate the efficiency of hGF-CM on tissue repair and engineering [20,36,37,38,39,40,41,42,43]. hGF-CM accelerated the cell migration, proliferation, and angiogenic functions of fibroblasts, keratinocytes, and endothelial cells [20,41]. The presence of neurotrophins, nerve growth factors, anti-inflammatory cytokines, such as IL-10 and transforming growth factor beta (TGFβ), in hGF-CM enables the protection of neuron-like NSC-34 cells from apoptosis, oxidative stress, and inflammation [40]. hGF-CM also promoted periodontal defect regeneration and bone formation in vivo [36,37]. Considering the history and previous publications, it seemed reasonable to examine the interaction between hGF-CM and DPSCs in an attempt to recapitulate pulp regeneration.

The aim of this study was to investigate the beneficial effects of hGF-CM on DPSC proliferation, migration, and differentiation, which are crucial elements in pulp regeneration.

## 2. Materials and Methods

### 2.1. Primary Culture of DPSCs and hGF

For dental pulp stem cell (DPSC) isolation, exfoliated deciduous teeth (from 6- to 12-year-old children) were extracted for clinical purposes and collected in the Pediatric Department of the Dental Hospital, Dankook University, under the approved guidelines approved by the Ethical Committee of the Institutional Review Board of Dankook University Dental Hospital (IRB number DKUDH 2019-10-001). All participants provided written informed consent. Primary cells were gathered according to previous literature [44,45,46,47,48]. 

After extracting each fresh tooth, it was kept in 5 mL Hanks’ balanced salt solution (HBSS, Welgene, Korea) and 1% penicillin-streptomycin (PS, Gibco, Thermo Fisher Scientific, Waltham, MA, USA) at 4 °C. Pulp was separated from the tooth, minced, and then dissociated in a digestive solution containing collagenase type I (Worthington Biochemical, Lakewood, NJ, USA) and dispase (Invitrogen, Carlsbad, CA, USA) for 1 h at 37 °C in a water bath. The suspension of dissociated cells was centrifuged at 1500 rpm for 5 min at room temperature. After removing the supernatant, growth medium (αMEM supplemented with 15% fetal bovine serum (FBS, Corning, NY, USA), 1% penicillin/streptomycin (PS, Thermo Fisher Scientific, MA, USA), 2 mM L-glutamine (GlutaMAXTM-1 100X, Gibco), and 0.1 mM L-ascorbic acid phosphate (Gibco)) was added to the cell pellet. This single-cell suspension was filtered with a 70 µm strainer (Falcon, BD Biosciences Discovery Labware, Bedford, MA, USA) and then centrifuged again under the above conditions. The final cell suspension was plated on a 60 mm cell culture dish (Falcon, Lincoln Park, NJ, USA) and incubated under the conditions of 5% CO_2_ and 95% humidity at 37 °C. The culture medium was changed after 48 h of initial incubation and then every 5 days thereafter. After 10 to 14 days, cells were subcultured when the cell confluence reached 80–90%, and cells at passages 4 to 10 were used for the following experiments.

**Figure 1:**
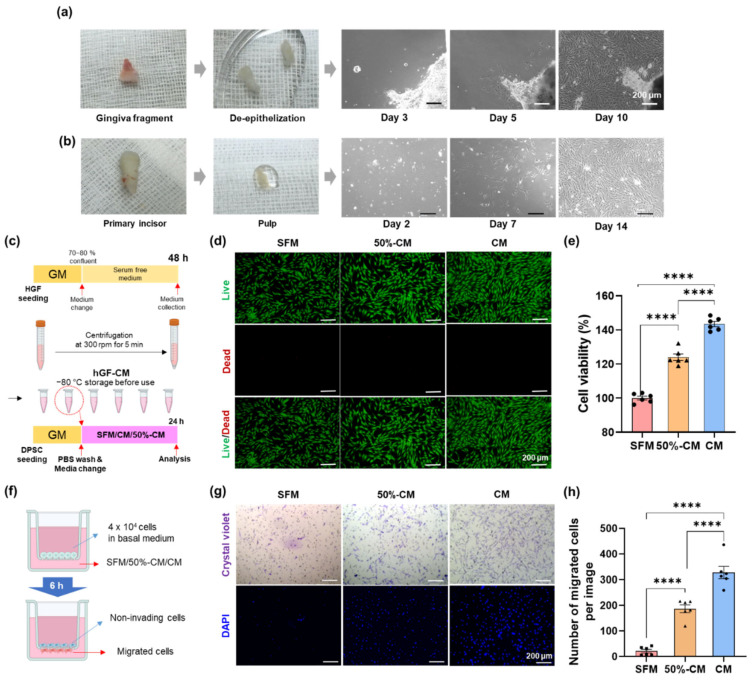
Effect of human gingival fibroblast-derived conditioned medium (hGF-CM) on the proliferation and migration of DPSCs. (**a**,**b**) Primary culture of hGF and hDPSC. (**a**) hGF started to spread out from de-epithelized gingiva masses at Days 3−5 after plating. It was highly proliferated and subcultured 10−14 days after seeding. (**b**) Primary DPSC isolated from pulp was observed at 16−24 h after seeding and then clustered on days 4−6. It was subcultured on Day 14 after seeding. Both hGF and hDPSC have a typical fibroblast-like appearance. (**c**–**e**) The effect of hGF-CM on proliferation. (**c**) Schematic images of the preparation for hGF-CM and the proliferation test on DPSCs to investigate the effect of hGF-CM. (**d**) Representative live/dead images of DPSCs 6 h after treatment with either hGF-CM, 50% hGF-CM, or SFM. (**e**) Quantified graphs of the viability of DPSCs measured by the CCK-8 assay. *n* = 6. (**f**–**h**) The effect of hGF-CM on migration. (**f**) Schematic image of the migration assay. A total of 4 × 10^4^ cells in 100 µL basal medium were seeded in the upper well, and the lower compartment contained 350 µL hGF-CM, 50% hGF-CM, or SFM. (**g**) Representative images of migrated DPSCs to the lower side of the membrane stained with crystal violet and DAPI after treatment for 6 h. (**h**) Cells were counted on the lower side of the membrane with DAPI images in 6 random fields. All data are represented as the mean ± SEM. One-way analysis of variance followed (ANOVA) by Tukey’s multiple comparisons test was used to detect statistically significant differences between groups. Represents **** *p* < 0.0001, scale bars represent 200 µm. SFM: Serum free medium, GM: Growth medium, CM: Conditioned medium, 50% CM: Conditioned medium diluted by 50% with serum free medium.

For the isolation of human gingival fibroblasts (hGFs), gingival fragments (from 13-year-old female patients) were obtained from surgical tooth extractions at the Oral and Maxillofacial Surgery Department, Dental Hospital, Dankook University. All participants provided written informed consent. After harvesting the gingival fragment, the fresh tissue was kept in 5 mL of HBSS and 1% PS. The gingival fragment was immersed in phosphate-buffered saline (PBS, Tech and Innovation, Chuncheon, Korea) containing 2% PS for approximately 1–2 min to reduce contamination risk and then washed in abundant 1X PBS to remove excessive PS. Blood vessels were trimmed away using scissors and forceps. After being submerged and dissociated in digestive solution with collagenase type I (2 mg/mL) and dispase II (4 mg/mL) at 37 °C for 1 h, the gingival epithelial layer was separated, cut into pieces approximately 1 × 1 mm in size, and then placed in a culture plate (60 mm, Falcon). The tissue was flooded with less than 2 mL growth medium (MEM) alpha modification, with L-glutamine, with ribo- and deoxyribonucleosides (HyClone Laboratories; GE Healthcare Life Sciences, Logan, UT, USA) supplemented with 10% fetal bovine serum (FBS, Corning), 2 mM L-glutamine (GlutaMAX^TM^-1 100X, Gibco), 100 mM non-essential amino acids solution (100X, Gibco), 55 µM 2-mercaptoethanol (1000X, Gibco), and 1% PS). The culture plate was incubated at 37 °C in a humidified atmosphere of 95% O_2_ and 5% CO_2_. The culture medium was changed after 48 h of initial incubation and then at least once every 48 h thereafter for 10 to 14 days. The colonies of primary cells were collected and subcultured. Then, the cells were detached by trypsin-EDTA and passaged when the cell confluence reached 80–90%, and passage 5 was used for the following experiments (Figure 1a,b).

### 2.2. Preparation of hGF-Conditioned Medium

Gingival fibroblasts at the 5th passage were cultured to 70–80% confluence in a 100 mm cell culture dish, and the hGFs were washed thoroughly 3 times with 1X PBS and then replenished with serum-free αMEM. After incubating at 37 °C in 5% CO_2_ for 48 h, the hGF-cultured medium was collected and centrifuged at 3000 rpm for 5 min at 4 °C to remove cell debris. The supernatant of the centrifuged medium was passed through 0.2 μm filters to obtain the final conditioned medium (CM). For the control medium, serum-free αMEM (SFM) was incubated for 48 h without cells under the same conditions and collected in the same way. All CM and SFM were subpackaged in 2 mL microtubes and stored at −80 °C for further use.

### 2.3. Cell Viability and Proliferation Assays

The effect of hGF-CM on the viability and proliferation of DPSCs was measured using a cell counting kit-8 assay (CCK-8, Dojindo, Kumamoto, Japan). In brief, DPSCs were seeded into 96-well plates at a density of 5 × 10^3^ cells/well and allowed to attach overnight. DPSCs were washed with 100 µL of 1X PBS twice before shifting to either CM, 50% CM (CM:SFM at a 1:1 ratio), or SFM. After 24 h of incubation, the medium was replaced with alpha MEM containing 10% CCK-8 solution in each well of the plate and incubated at 37 °C for 2 h. Then, a microplate reader (Thermo Fisher Varioskan^TM^ LUX, Waltham, MA, USA) recorded the absorbance of each well at 450 nm. The cell viability in the control group (normal growth medium group) was considered 100%, and the percentage values for the experimental groups were calculated. The survival of cells was also confirmed by live/dead staining (0.5 µM calcein-AM and 2 µM ethidium homodimer-1 solutions, Thermo Fisher, USA), and images were taken using a fluorescence microscope (IX71, Olympus, Tokyo, Japan).

### 2.4. Cell Migration Assay

To detect the effect of hGF-CM on the migration ability of DPSCs, 4 × 10^4^ DPSCs in 100 μL serum-free αMEM were plated in the upper chamber of a Transwell with 8-μm pores (Corning), and the lower chamber (24-well plate) was injected with 350 μL either hGF-CM, 50% hGF-CM or SFM. After incubating for 6 h at 37 °C and 5% CO_2_, the Transwells were fixed with 4% paraformaldehyde (Merck) for 15 min and stained with 2.5 mg/mL crystal violet solution (Sigma-Aldrich, St Louis, MO, USA) in 20% methanol for 30 min. After washing the cells with PBS, the upper surface of the Transwell was cleaned with cotton swabs. Images of stained cells were taken with an inverted microscope (IX71, Olympus, Tokyo, Japan). In addition, DPSCs were stained with DAPI, and the number of migrated cells was counted by ImageJ (version 1.53t).

### 2.5. Antioxidative Stress Assay

To mimic the microenvironment of oxidative stress detected in the transplantation site, hydrogen peroxide (H_2_O_2_), which is a well-known oxidative stress inducer, was used.

To determine the proper concentration of H_2_O_2_, DPSCs (5 × 10^3^ cells/well, 96-well plates) were exposed to different concentrations of H_2_O_2_ (0, 100, 150, and 200 µM) in serum-free αMEM. After 12 h, 100 µL of CCK-8 solution diluted with a-MEM at a ratio of 1:10 was added and then incubated at 37 °C for 2 h. The absorbance of the orange product was measured at 450 nm using a microplate reader and calculated as we mentioned in cell viability and proliferation assays.

The live/dead assessment of cells was also carried out. The cells were incubated in alpha MEM containing 2 μM calcein-AM and 4 μM PI (Invitrogen) for 20 min and then observed by a fluorescence microscope (IX71, Olympus, Tokyo, Japan). As a negative control, cells treated with the same amount of DW used for H_2_O_2_ treatment were evaluated. Based on this result, 150 µM was selected for further experiments.

To investigate the antioxidative effect of hGF-CM, DPSCs were treated with 150 µM H_2_O_2_ for 12 h, and the plates were washed twice with PBS. Either hGF-CM, 50% hGF-CM, or SFM was applied and cultured for an additional 12 h. After that, the CCK8 assay and live/dead imaging were performed as described above.

### 2.6. Osteogenic Differentiation Assays

To assess the effect of hGF-CM on osteogenesis, OCM, OhCM, and OFM were prepared, which are hGF-CM, heated CM (CM heated at 95 °C for 30 min to denature the proteins), or SFM diluted with odontogenic medium (αMEM, 10% FBS, 1% PS, 10 mM β-glycerophosphate, 100 nM dexamethasone (Sigma), and 50 μg/mL ascorbic acid) at a 1:1 ratio. DPSCs were plated on a 24-well plate (1 × 10^4^ cells/well) and incubated in growth medium overnight. The following day, the medium was replaced with OCM, OhCM, or OFM and changed every 3 days.

Odontogenic gene expression was evaluated on days 3, 7, and 14 by qRT—PCR. In brief, total RNA was extracted using Ribospin^TM^ (cat. no. 304-150; GeneAll Biotechnology, Seoul, Korea) according to the manufacturer’s instructions. Total RNA (1 µg) was used for reverse transcription with Accupower RT premix (K-2043, Bioneer, Daejeon, Korea), and qRT-PCR was performed following the 2X SensiMix SYBR Hi-ROX Mastermix (QT-605-05, Bioline, London, UK) protocol on a StepOnePlus real-time PCR system (Applied Biosystems, Foster City, CA, USA). The gene expression levels were analyzed and normalized to GAPDH. The fold change in gene expression was analyzed by the 2^−ΔΔCT^ method. Each primer sequence is listed in Table 1.

The differentiation efficiency was evaluated by alkaline phosphatase (ALP) staining on Days 3 and 7 and Alizarin Red S staining (ARS) on Days 14 and 21. In brief, the differentiated cells were fixed with 4% paraformaldehyde at room temperature for 15 min, and then ALP solution (SIGMAFAST^TM^ BCIP^®^/NBT tablet dissolved in 10 mL distilled water) was applied to check the ALP activity for 1 h at 37 °C. To assess mineralization, Alizarin Red S reagent (40 mM, pH = 4.2) was applied for 30 min after fixation. After washing with distilled water, images of the stained cells were taken via light microscopy at 100× magnification. For quantification of ARS staining, the stained mineral deposits were dissolved in a 10% (*w*/*v*) cetylpyridinium chloride (CPC, Sigma—Aldrich, MO, USA) solution on a rocking shaker for 30 min at room temperature. The optical density of ARS extracts was measured at 562 nm by a microplate reader.

### 2.7. Proteomic Analysis

The hGF-CM was prepared as mentioned above and concentrated ten times using the centrifugal concentrator (100K cutoff, Amicon^®^ Ultra-15 Centrifugal Filter Unit, Millipore). The proteomics was commercially commissioned by E-biogen (E-biogen. Inc., Seoul, Korea). In brief, the concentration of protein was quantified using the Pierce BCA Protein Assay Kit (Thermo Fisher Scientific, Waltham, MA, USA). The peptides were eluted through reduction, digestion, and desalting using filter-aided sample preparation (FASP) on Microcon 30K centrifugal filter device (Millipore, Billerica, MA, USA). Samples were analyzed on an UltiMate 3000 RSLC nano LC system (Thermo Scientific) coupled to a Q Exactive mass spectrometer (Thermo Scientific). MS/MS data were converted to mzXML using MSConvert for searching in Andromeda of MaxQuant (version 1.5.8.3). Information on the recognized peptides and proteins was aligned using the mass of charge state, retention time, and peak. The data provided by E-biogen were sorted using EXDEGA (E-biogen) to have a coverage value over 10%, which analyzed the GO term and KEGG pathway using DAVID.

### 2.8. mRNA Sequencing

The total RNA of DPSCs cultured in OCM, OhCM, and SFM for 7 days was isolated using Ribospin^TM^ (cat. no. 304-150; *GeneAll* Biotechnology) according to the manufacturer’s protocol. RNA sequencing was commercially commissioned by E-biogen (E-biogen. Inc., Seoul, Korea).

Significant genes in the OCM group compared to SFM were selected with three conditions: fold-changes > 2.0, normalized data (log2) > 4, and *p*-value < 0.05 using ExDEGA software (v1.6.7, E-biogen) with raw data provided by E-biogen. The enriched functional annotation terms were analyzed with the above data by DAVID and visualized using ExDEGA GraphicPlus (E-biogen). A heatmap of hierarchical clustering was generated using MeV version 4.9.0. The RNA-seq data in this study are available at the NCBI Gene Expression Omnibus with accession number GSE 201524.

### 2.9. Inhibition Assay

SHED (1 × 10^4^ cells/well) were plated in 24-well plates in growth medium and incubated overnight, allowing cell attachment. On the following day, the medium was changed to OCM, and the two inhibitors, U0126 (Sigma Aldrich) and RepSox (Tocris, Ellisville, MO, USA), were treated individually. As a control group, DMSO, which was used to dilute the inhibitors, was used as the treatment without inhibitors. The media were refreshed with inhibitors or DMSO every 3 days for 14 days. Odontogenic differentiation-related gene expression levels (DMP1, RUNX2, and OCN) were measured on Days 3 and 7, and ALP activity and mineralization were confirmed by ALP and ARS staining on Days 7 and 14, respectively.

### 2.10. Statistical Analyses

Data were analyzed using GraphPad Prism 8 software (San Diego, CA, USA) and are shown as the mean  ±  standard deviation. One-way analysis of variance followed by Tukey’s multiple comparisons test was used to detect statistically significant differences between groups. *p* < 0.05 was considered significant.

## 3. Results

### 3.1. Effect of hGF-CM on DPSC Proliferation

The effect of hGF-CM on DPSC proliferation was assessed after 6 h of treatment. Treatment with hGF-CM and 50% CM significantly increased DPSC proliferation compared to SFM. Treatment with 100% CM yielded the highest level of cell proliferation, approximately 24% higher than SFM, and 50% CM increased cell proliferation by 40% compared to SFM, indicating that hGF-CM has a high capacity for enhancing cell proliferation in a dose-dependent manner. The live/dead assay results were consistent with the CCK-8 assay (Figure 1c–e).

### 3.2. Effect of hGF-CM on DPSC Migration

SFM did not seem to induce cell migration, as few cells that had migrated to the bottom surface of the membrane were observed (21 ± 16 cells). In contrast, hGF-CM-treated DPSCs showed a significantly higher migration capacity than cells treated with SFM (*p* < 0.001). A total of 100% CM induced the greatest migration, with an average of 327 ± 59 cells, and 50% CM was not as efficient as CM, with 188 ± 37 cells, indicating that hGF-CM is also highly effective for cell migration in a dose-dependent manner, similar to the proliferation effect (Figure 1f–h).

### 3.3. Protective Effect of hGF-CM against H_2_O_2_-Induced Cell Death in DPSCs

**Figure 2:**
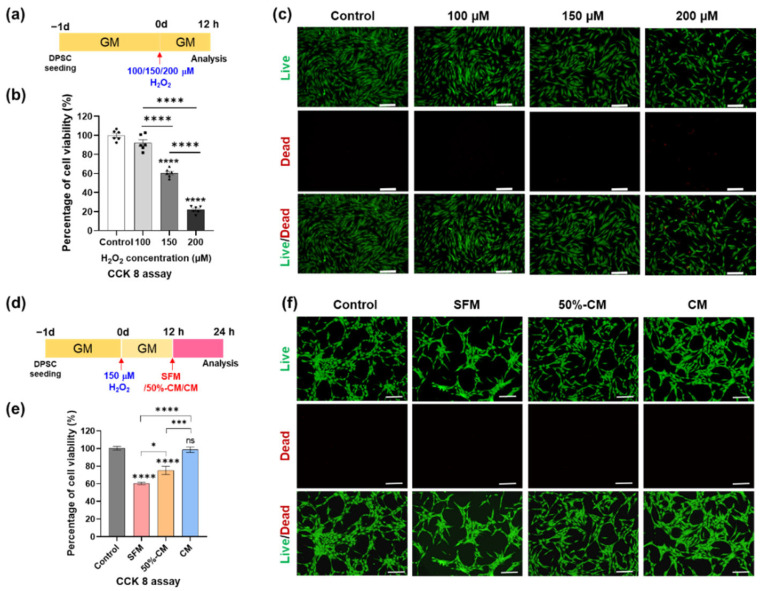
Treatment with hGF-CM improves DPSC viability after H_2_O_2_ exposure. (**a**–**c**) DPSC viability exposed to different concentrations (100, 150, and 200 µM) of H_2_O_2_ for 24 h. (**a**) Schematic image of the timetable for the viability test depending on the H_2_O_2_ concentration. (**b**) Quantified results determined by CCK-8 analysis. (**c**) Representative live/dead images showing the oxidative stress effects induced by H_2_O_2_ treatment on DPSCs. (**d**–**f**) DPSC viability cultured in either CM, 50% CM, or SFM for another 12 h after exposure to 150 µM H_2_O_2_ for 12 h (**d**) Schematic image of the timetable. (**e**) Cell viability measured by CCK-8 analysis. (**f**) Representative live/dead images of DPSCs confirmed the antioxidative stress effect of hGF-CM. All data are represented as the mean ± SD. One-way analysis of variance followed (ANOVA) by Tukey’s multiple comparisons test was used to detect statistically significant differences between groups. Represents * *p* < 0. 05, *** *p* < 0. 001, **** *p* < 0.0001, ns = not significant, scale bar = 200 µm. SFM: Serum free medium, GM: Growth medium, CM: Conditioned medium, 50% CM: Conditioned medium diluted by 50% with serum free medium.

Although H_2_O_2_ can induce a beneficial microenvironment for some basic cellular activities, such as proliferation and migration, at low levels, high levels of H_2_O_2_ can activate the pathological microenvironment. To investigate the concentration of H_2_O_2_ that damages the viability of DPSCs, DPSCs were treated with various concentrations of H_2_O_2_ (100, 150, and 200 μM) for 12 h and then analyzed by CCK-8 and live/dead assays. The results showed that the viability of DPSCs decreased in a dose-dependent manner in response to H_2_O_2_ treatment. Based on the CCK-8 assay results, treatment with 150 μM H_2_O_2_ decreased cell viability by approximately 50%; therefore, this concentration of H_2_O_2_ was selected for subsequent experiments (Figure 2a–c).

To evaluate the recovery effect of hGF-CM in H_2_O_2_-induced ROS conditions, DPSCs were treated with 150 μM H_2_O_2_ for 12 h and applied to SFM, 50%-CM, or CM after washing with PBS twice and then incubated for the next 12 h. While the SFM group showed 60.56% ± 3.42% cell viability, the 50%-CM and CM groups showed 75.20% ± 11.62% and 98.72% ± 7.21% viability (almost the same as the control), proving the recovery of cell viability after oxidative stress. (Figure 2d–f). The results of the live/dead assay were consistent with the CCK8 assay results (Figure 2b). In conclusion, it was confirmed that CM (even with a 50% amount) had great potency to recover viability from cell damage after ROS conditions induced by H_2_O_2_.

### 3.4. hGF-CM Enhances DPSC Odontoblast Differentiation Capacity

**Figure 3:**
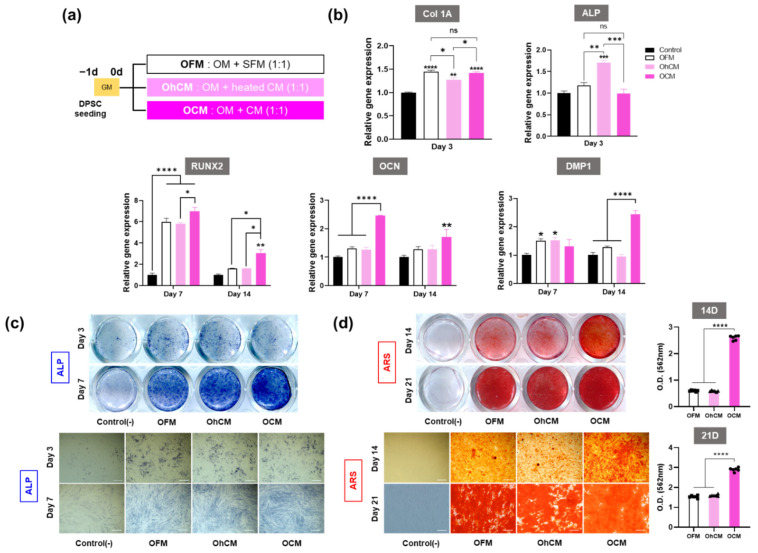
hGF-CM potentiates SHED odonto/osteogenic differentiation (**a**) Schematic diagram showing the schedule and groups of experiments. (**b**) Relative odonto/osteogenic gene expression levels quantified by qRT-PCR, with early markers at Day 3 and late markers at Days 7 and 14. (**c**) Representative images of alkaline phosphatase (ALP) activity analyzed by ALP staining at Days 3 and 7 and (**d**) mineralization analyzed by Alizarin Red staining at Days 14 and 21 after culturing in OFM, OhCM, or OCM. The graphs show the quantification of mineralization at Days 14 and 21. (**c**) All data are represented as the mean ± SD. All data are represented as the mean ± SD. One-way analysis of variance followed (ANOVA) by Tukey’s multiple comparisons test was used to detect statistically significant differences between groups. Represents * *p* < 0.05, ** *p* < 0.01, *** *p* < 0.001, **** *p* < 0.0001, ns = not significant, scale bar = 200 µm. OFM: hGF-CM diluted by 50% with odontogenic medium, OhCM: heated CM (CM heated at 95 °C for 30 min to denature the proteins) diluted by 50% with odontogenic medium, OCM: SFM diluted by 50% with odontogenic medium.

We performed odontogenic induction of DPSCs in odonto/osteogenic differentiation medium diluted in CM, heated CM, or SFM at a 1:1 ratio (Figure 3a). Odontogenic differentiation capacity was evaluated through the gene expression of odontoblast markers (qRT—PCR), ALP activity (ALP staining), and mineralization (ARS staining).

qRT-qPCR data revealed that hGF-CM significantly increased the transcript levels of odontoblast-specific markers, especially late markers, compared to the control group (Figure 3b). At 3 days, Col1A, the early marker of odontoblasts, showed higher expression in all the groups compared to the control, while there was no significant difference in OCM groups compared to the OFM groups. In addition, the ALP gene was highly expressed only in OhCM, not in OCM and OFM. In conclusion, even if some groups showed higher expression in early markers of odontogenesis, the difference was less than two-fold and, consistent with this, it was difficult to distinguish the difference between the groups in ALP activity by ALP staining on Day 3 (Figure 3c). The promotive effect of CM on odontogenic differentiation was seen from Day 7 when relevant gene expression levels (RUNX2, OCN, and DMP1) in the OCM group were upregulated compared with those in the OhCM and OFM groups (*p* < 0.05) (Figure 3b–d). In particular, the odontoblastic-related gene expression DMP-1 was greatly upregulated on Day 14 when DPSCs were cultured in OCM, almost two-fold compared to OhCM and OFM. The results of ALP activity and mineralization were consistent with the upregulation of relevant gene expression. Under OCM treatment conditions, ALP staining became stronger on Day 7, and the area of positive ARS staining was larger on Days 14 and 21 (Figure 3c,d). Consistently, the quantitation levels of calcium deposition were 4 and 2 times higher in the OCM group on Days 14 and 21, respectively (Figure 3d). Based on these results, we conclude that hGF-CM can induce earlier mineralization and promote the mineralization level in the odontogenesis of DPSCs.

### 3.5. Differential Protein Expression in hGF-CM

**Figure 4:**
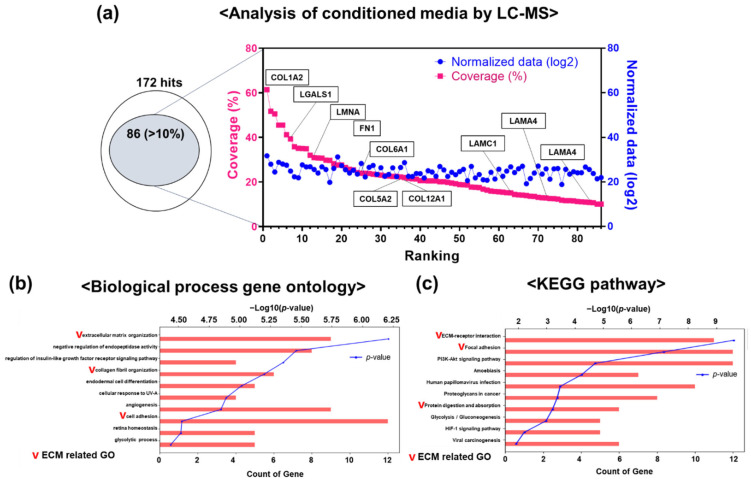
LC-MS analysis of conditioned media from HGF. (**a**) From LC-MS analysis of ten times concentrated hGF-CM, 172 hits were detected. Among them, over 10% coverage was found from 85 hits, revealing extracellular matrix (ECM) proteins such as collagen and laminin. (**b**,**c**) Gene ontology analysis using DAVID revealed ECM-related biological GO terms and pathways.

LC-MS-based proteomics with hGF-CM was conducted to determine which proteins are sufficient in hGF-CM and effective for odontogenesis, and as a result, a considerable number of ECM components were identified. Among them, 85 hit components were selected and analyzed again, and biological processes and pathways related to ECM were detected (Figure 4a−c). The phenomenon that there are many ECM-related molecules from these kinds of CMs is consistent with the known effects of ECM components on increasing biomineralization in differentiation (especially collagen, etc.). Detectable growth factors other than ECM described in other literature were not detected, which is thought to be due to the detection limit of LC-MS.

### 3.6. Signaling Pathway Involved in Enhancing the Capacity of hGF-CM in Odonto/Osteogenesis

**Figure 5:**
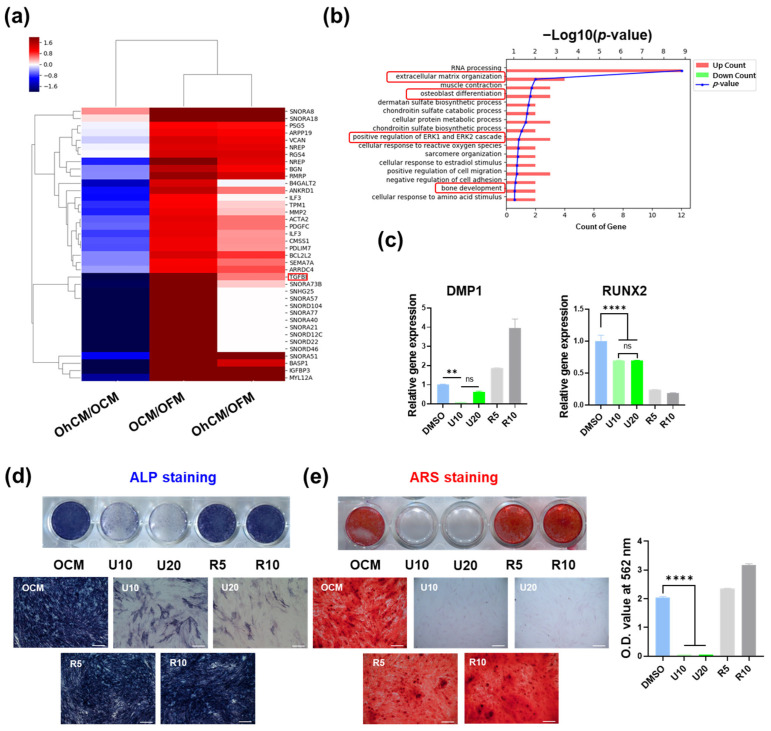
RNA-seq data analysis of DPSCs cultured in OCM, OhCM, and OFM and inhibition assay (**a**,**b**) Twenty-eight genes among a total of 51,219 significantly expressed (fold change > 2, normalized data (log2) > 4, *p* < 0.05) in DPSCs cultured OCM compared with OFM were selected for gene expression heatmap and GO (gene ontology) term analysis. (**a**) Heatmap of hierarchical clustering. (**b**) Functional enrichment analysis. The distribution of gene ontology (GO) terms of DEGs was annotated in the biological ontology category (BP). (**c**,**d**) ERK inhibitor (U160, 10 and 20 µM) and TGF-β receptor inhibitor (RepSox, 5 and 10 µM) were treated in OCM every 3 days for 7 days during osteogenic induction. (**c**) Relative gene expression related to odonto/osteogenesis (DMP1, RUNX2, and OCN) analyzed by qRT-PCR on Day 7. (**d**) Representative images stained by ALP on Day 7 and (**e**) stained by Alizarin Red S staining on Day 14. All data are represented as the mean ± SD. One-way analysis of variance (ANOVA) followed by Tukey’s multiple comparisons test was used to detect statistically significant differences between groups. Represents ** *p* < 0.01, **** *p* < 0.0001, ns = not significant. BP: biological process. U10 (U0126, 10 µM), U20 (U0126, 20 µM), R5 (RepSox, 5 µM), R10 (RepSox, 10 µM).

To analyze the effect of hGF-CM on osteogenesis in detail, mRNA sequencing was performed with DPSCs cultured for 7 days in OCM, OhCM, and OFM. A heatmap of hierarchical clustering was generated with 38 differentially expressed genes in OCM compared with OFM (*p* < 0.05). The results showed that only some of the genes that were significantly increased in OFM compared to OFM were increased in OhCM and downregulated compared to OCM (Figure 5a). However, among the most enriched GO terms in those genes were terms related to odonto/osteogenesis, such as extracellular matrix organization, osteoblast differentiation, positive regulation of ERK1, the ERK2 cascade, and bone development (Figure 5b). To investigate which signaling pathway is involved in the enhancement of odonto/osteogenesis by hGF-CM, the clue was explored based on mRNA-seq data. As a result, ERK inhibitor (U0126) and TGF-β receptor inhibitor (RepSox) were selected as candidates via GO term analysis and DEG data, respectively. The changes in odontogenic gene levels, ALP activity, and mineralization were compared with/without inhibitor treatment on Days 7 and 14. As we expected, odontogenic gene expression was downregulated compared to that in the nontreated group, and DAMP1 decreased in a dose-dependent manner in the U0126-treated group. The gene expression changes in the RepSox-treated group showed inconsistent patterns. DAMP1 and OCN were significantly upregulated, and RUNX2 levels were decreased even more than in the U0126-treated group (Figure 5c). Consistent with the qRT—PCR results, while the RepSox-treated group showed similar or slightly increased ALP activity on Day 7 and mineralization on Day 14, the U0126-treated group revealed significantly decreased ALP activity on both days (Figure 5d,e). Collectively, the odonto/osteogenic effect of hGF-CM is highly involved in the ERK pathway.

## 4. Discussion

Cell-based pulp regeneration has been considered a promising method since many stem/progenitor cell sources, such as DPSCs and hGFs, have been reported to have the potential for pulp regeneration, as they are able to differentiate into odontoblast-like cells [16,18,49,50,51]. Based on this account, various studies have been conducted using cell-based approaches to obtain ideal pulp-dentin regeneration [24,50,52,53,54,55]. However, cell-based approaches are faced with two principal challenges: a proper, reliable cell source and a favorable microenvironment [16,50].

As mentioned above, DPSCs have been considered a potential candidate for the generation of pulp-like complexes due to their high proliferation rate, multiple lineage differentiation capacities, accessibility, and lack associated ethical concerns [17,18,49]. Similarly, hGF represents the most accessible, abundant source for stem cells with multipotency and potent immunomodulatory/anti-inflammatory capacities [20,32,35,40]. In addition, conditioned medium obtained from cultured hGFs has been extensively investigated as a regenerative therapeutic for the treatment of a wide range of pathological conditions and diseases, such as skin wound repair and injured nerve, tendon, periodontal, and bone defect regeneration [20,24,31,36,37,40]. In the present study, we investigated the effects of hGF-CM on proliferation, migration, antioxidative durability under H_2_O_2_-induced apoptosis, and odontoblastic differentiation of DPSCs to prove its efficacy for pulp tissue regeneration.

Cell growth is one of the most critical factors in tissue regeneration. As shown in Figure 1a–c, our results revealed that the proliferation rate was increased by 40% in 100% hGF-CM and 24% in 50% CM compared to SFM at 1 day after application, which indicated the positive effect of hGF-CM on cell proliferation. This result corresponded with previous studies of hGF-CM on human keratinocytes and fibroblasts. The addition of hGF-CM significantly increased the proliferation of both keratinocytes by 30% (*p <*  0.0001) and fibroblasts by 16% (*p <*  0.0001) after 24 h of treatment with hGF-CM compared to the negative control [20]. Likewise, mixed-culture CM treatment induced a three-fold increase in squamous cell carcinoma-25 cell proliferation [56].

In tissue regeneration strategies, cells that are capable of forming new tissue come from two main sources: transplanted and endogenous stem cells. Therefore, the homing of MSCs is the critical first step in the process of induced regeneration [57,58]. Many studies have demonstrated that extracellular vesicles and supernatants derived from cultured hGF contain chemoattractants that regulate cell mobility [20,35,40,41,56,59]. hGF-CM significantly elevated the migratory ability of human keratinocytes and foreskin fibroblasts in two-dimensional wound healing assays [20]. In the same way, scratched gaps of carcinoma cells closed 1.6 times faster than the control group within 96 h when treated with both hGF-CM or mixed-culture CM [56]. The results of the Transwell chamber migration assay demonstrated that the modified hGF culture supernatant significantly increased the number of migrating human umbilical vein endothelial cells compared with the negative control [41]. In agreement with previous studies, hGF-CM attracted significant migration of cells (approximately three times) compared to SFM. This greater migratory activity in the presence of hGF-CM indicated that it is able to attract endogenous stem cells in the process of pulp regeneration.

One of the major limitations to the use of MSCs in clinical applications is the low survival of transplanted cells due to an unfavorable microenvironment [60]. A multitude of environmental signals, including oxidants or stimulators of intracellular generation of reactive oxygen intermediates (ROIs), can trigger apoptotic cell death. To mimic the microenvironment of high oxidative stress detected in transplantation sites, DPSCs were exposed to 150 µM hydrogen peroxide (H_2_O_2_). Treatment with hGF-CM significantly reduced apoptotic cell death and showed cell viability levels that were similar to those of the untreated H_2_O_2_ group. This result showed a similar trend to a previous study that reported that hGF-CM inhibits the apoptosis process in injured neurons [40]. It can be inferred that hGF-CM has the potential to improve tissue regeneration through the enhancement of cell viability in the rough microenvironment of the injured site.

The critical step of pulp regeneration is the differentiation into odontoblasts and, in turn, the formation of new dentin. To evaluate the effect of hGF-CM on differentiation capacity, DPSCs were cultured under hGF-CM conditions. Although abundant proteins and growth factors were found in CM, essential components for cell culture in culture media would have been mostly consumed [43,61]. To supply minimum nutrients for cell growth and simulate the odontogenic environment, CM was diluted with normal odontogenic differentiation media by 50%. The effect of hGF-CM on odonto/osteoblastic differentiation of DPSCs was determined by staining ALP activity, assessing mineralization, and analyzing the expression levels of odontogenic genes, such as Col1A, ALP, Runx2, DMP-1, and OCN. Mandeep et al. revealed that calvarial osteoblasts decreased ALP activity during the first 3 days of coculture with hGF-CM [38].

Consistent with this finding, hGF-OM-treated DPSCs showed decreased levels of ALP activity and gene expression on Day 3 of our study. The suppression of odontogenic ALP expression during the first 3 days may be balanced by the higher proliferation of DPSCs under hGF-CM conditions [38,62]. The odontogenic differentiation capacity of DPSCs in hGF-CM was elevated at late stages when the levels of odontogenic gene expression (DMP-1, RUNX2, and OCN) and alizarin red staining increased from Day 7. This result was similar to Kang’s study, which reported that DPSCs cultured in hGF-CM, even with 40% mineralization additives, induced more mineralization than the control [39]. Moreover, 3D-PLA scaffolds enriched with hGF-derived CM showed a better osteogenic capacity and are considered to play key roles in the induction of the osteogenic process and in bone regeneration [37].

This odontogenic effect of hGF-CM could not be found in the case of OCM (CM containing denatured proteins from heating), and its odontogenesis level was comparable to that of SFM. This result indicates that growth factors and cytokines released from hGF may be greatly effective in facilitating odontogenic differentiation.

Various signaling pathways are involved in osteo/odontogenesis, including ERK1/2 (extracellular signal-regulated kinases), Wnt/β-catenin, BMP/Smad, and Hedgehog [63]. We investigated which specific signaling pathway among them is most intensely activated by hGF-CM. RNA sequencing analysis initially revealed that differentially expressed genes (DEGs) in DPSCs cultured in OCM are highly related to odonto/osteogenesis. Then, GO term analysis confirmed that these genes are involved in the ERK pathway. Even though the ERK signaling pathway plays a key role in a wide variety of cellular processes, it has been reported in various situations that inhibition of ERK activity blocks osteogenesis in adult human MSCs [64], mouse MC3T3-E1 preosteoblastic cells [65], and human stem cells from the apical papilla (SCAP) [66], indicating that the ERK pathway plays a crucial role in osteo/odontogenesis. Consistent with this notion, our inhibitor study proved that hGF-CM enhanced odontogenetic capability via the ERK pathway.

The therapeutic effect of hGF-CM is mediated, at least partly, by secreted factors that promote cell differentiation, stimulate the migration of nearby stem cells, protect cells from apoptosis, facilitate angiogenesis, and enhance odontoblast differentiation. However, the efficacy of hGF-CM on pulp regeneration still needs further investigation with a relevant in vitro model. Moreover, although we studied and showed the possibility of hGF-derived CMs as a candidate for pulp regeneration in this study, we cannot clarify to say which dental stem cell-derived CM is the best for pulp regeneration. Hence, a comparison of CM derived from various dental stem cells in pulp regeneration needs to be investigated with appropriate in vivo models, such as the inflamed pulp model [30], in further studies. In addition, we only tested the effect on DPSC isolated from young donors, so it should be demonstrated in future studies how effective it is in adult-derived DPSC.

**Figure 6:**
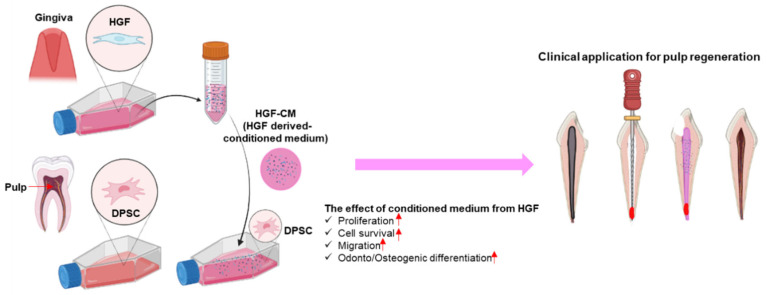
Schematic diagram of the effects of hGF-CM on DPSC culture and differentiation. hGF-CM increased cell proliferation and progenitor cell recruitment, recovered cell viability from oxidative stress, and enhanced osteo/odonto differentiation. Hence, hGF-CM can be a promising candidate for pulp regeneration therapy in the clinic.

Although there is no doubt of their clinical effectiveness, current treatment methods for necrotic pulp are far from the concept of ideal pulp regeneration. Root canal treatment involves the mechanical and chemical cleansing of the canal followed by obturation with bioinert materials. Other techniques are only applicable to immature teeth, as they rely on the innate healing capability of the remaining vital pulp and periapical tissues. Attempts to revolutionize therapeutic dental pulp regeneration by building a system that solely depends on external agents (stem cells and scaffolds) are in the development stage, with approaches from various aspects being tested in laboratories. The current study, with the incorporation of a conditioned medium as the key mechanism, contributes to the full realization of ideal pulp regeneration (Figure 6).

## 5. Conclusions

The results of the present study suggest that hGF-CM increases the proliferation and migration ability of DPSCs in a dose-dependent manner and facilitates the odontogenesis of DPSCs through ERK pathway signaling. Although there is a lack of in vitro evidence, DPSCs and hGF-CM could be a promising combination for pulp regeneration in the future.

## Figures and Tables

**Table 1:** Primer sequences.

	Forward Primer (5′-3′)	Reverse Primer (5′-3′)
COL1A	5′-CGTGACCAAAAACCAAAAGTGC-3′	5′-GGGTGGAGAAAGGAACAGAAA-3′
ALP	5′-ACACCTTGACTGTGGTTACT-3′	5′-CCTTGTAGCCAGGCCCGTTA-3′
DMP1	5′-GGACGGCTCTGAGTTCGA-3′	5′-TGGGTTTCCCTGCTGTTG-3′
OCN	5′-AGACTCCGGCGCTACCTCAACAAT-3′	5′-CAGCTGTGCCGTCCATACT-3′
RUNX2	5′GCTTCATTCGCCTCACAAAC-3′	5′-GTAGTGACCTGCGGAGATTAAG-3′
GAPDH	5′-GAGCATCTCCCTCACAATTT-3′	5′-GGGTGCAGCGAACTTTAT-3′

## Data Availability

The RNA-seq data in this study are available at the NCBI Gene Expression Omnibus with accession number GSE 201524.
